# Melatonin Signaling and Its Modulation of PfNF-YB Transcription Factor Expression in *Plasmodium falciparum*

**DOI:** 10.3390/ijms140713704

**Published:** 2013-07-01

**Authors:** Wânia Rezende Lima, Anthony A. Holder, Célia R. S. Garcia

**Affiliations:** 1 Departamento de Fisiologia, Instituto de Biociências, Universidade de São Paulo, São Paulo 05508900, Brazil; E-Mail: wrlima@ib.usp.br; 2 Division of Parasitology, MRC National Institute for Medical Research, The Ridgeway, Mill Hill, London NW7 1AA, UK; E-Mail: aholder@nimr.mrc.ac.uk

**Keywords:** melatonin, malaria, signaling modulation, *Plasmodium*

## Abstract

Malaria is one of the most severe tropical infectious diseases. More than 220 million people around the world have a clinical malaria infection and about one million die because of *Plasmodium* annually. This parasitic pathogen replicates efficiently in its human host making it difficult to eradicate. It is transmitted by mosquito vectors and so far mosquito control programs have not effectively eliminated this transmission. Because of malaria’s enormous health and economic impact and the need to develop new control and eventual elimination strategies, a big research effort has been made to better understand the biology of this parasite and its interactions with its vertebrate host. Determination of the genome sequence and organization, the elucidation of the role of key proteins, and cell signaling studies have helped to develop an understanding of the molecular mechanisms that provide the parasite’s versatility. The parasite can sense its environment and adapt to benefit its survival, indeed this is essential for it to complete its life cycle. For many years we have studied how the *Plasmodium* parasite is able to sense melatonin. In this review we discuss the melatonin signaling pathway and its role in the control of *Plasmodium* replication and development.

## 1. Introduction

Malaria is caused by protozoan parasites of the genus *Plasmodium* that have impacted survival and driven human evolution throughout history. Whilst *Plasmodium* parasites infect some reptiles, birds and mammals, including primates, it is accepted that 10,000 years ago malaria impacted human survival during the beginning of agriculture [1]. Human malaria originated in Africa and extended to all other populated continents, but nowadays it is predominantly located in tropical regions, comprising 104 malaria-endemic countries according to the World Health Organization (WHO) database [2]. There are five species that naturally infect humans, of which *P. falciparum* and *P. vivax* are most prevalent. More than 220 million people suffer clinical malaria annually with over one million deaths, largely due to *P. falciparum* infection.

The human host is infected by the bite of an *Anopheles* mosquito, which delivers the sporozoite form of the parasite. Sporozoites migrate to the liver and differentiate into multinucleate exoerythrocytic schizonts, which then release merozoites that initiate the asexual blood stage of development. The merozoite invades a red blood cell (RBC) to commence intracellular development through the uninuclear ring and trophozoite stages that precede nuclear division in the schizont stage. The multinucleate schizont is formed and then division of the cytoplasm in late schizogony (the segmenter stage) forms new merozoites that are released to invade fresh RBC and continue the cycle [3]. This intraerythrocytic cycle in mammals takes approximately 24, 48 or 72 h depending on the parasite species and the host. For example in *P. falciparum*, one ring stage form may replicate and differentiate into 16 to 32 daughter merozoites [4,5] in one asexual cycle that lasts about 48 h. Schizont maturation and merozoite release occur relatively synchronously resulting in periodic fever, and further physiological complications as the disease evolves, including headaches, nausea and vomiting, anemia, renal failure [6], respiratory distress and in a few cases, cerebral malaria [7,8]. Transmission back to a mosquito is achieved by some intracellular parasites differentiating into male and female gametocytes that, if taken up in a blood meal, differentiate into male and female gametes in the mosquito gut allowing fertilization and infection of the insect. The periodicity and synchronicity of the asexual cycle is modulated *in vivo* according to the circadian rhythm of the host. For example the temperature cycle of the host [9] or the timing of the light-dark cycle [10] can be manipulated in experimental systems to change the pattern of development of the parasite.

The need to obtain insights into how to control or eliminate *Plasmodium* infection has promoted many researchers to try and understand better the parasite’s molecular machinery. Genome sequencing has provided tools to study and analyze the parasite’s biochemistry and physiological mechanisms. The *P. falciparum* genome sequence was determined ten years ago [11] since when many protein families have been discovered and characterized [12,13]. Studies on the importance of signaling pathways, gene regulation, and the mode of action of antimalarial drugs have contributed enormously to our understanding of how the parasite machinery works [14].

Melatonin (*N*-acetyl-5-methoxytryptamine) is a tryptophan-derived metabolite that participates in several physiological activities, not only synchronizing the circadian rhythm, but also playing a role in sleep, free radical scavenging, immune-regulation and as an antibacterial agent [15–19]. In vertebrates, melatonin has also been shown to regulate transcription factors, phosphorylation of cAMP-responsive element binding protein, and c-Fos expression [20]. Melatonin is also found in plants and unicellular organisms [21–24]. In this review we focus on the role of melatonin in the intraerythrocytic cycle of *Plasmodium* development, and highlight its potential to be targeted to develop antimalarial drugs.

## 2. Melatonin Signaling: Calcium and cAMP Generation in *P. falciparum* and *P. chabaudi*

Melatonin signaling controls a variety of physiological processes, some of which are influenced by the light/dark circadian cycle [25]. The circadian cycle is the period of one day (24 h) in which to complete activities of the biological cycle of an organism. One function of this system is adjustment of the biological clock, sleep and appetite control. A pacemaker, the suprachiasmatic nucleus (SCN), referred to as a master clock in mammals connects to a hormonal network to orchestrate the circadian rhythm [25–28]. Undoubtedly, the melatonin hormone is part of this network. Melatonin, plays a role in the circadian cycle of malaria parasite development, and has been shown to modulate the asexual cycle of both *P. falciparum* and *P. chabaudi* [29,30]. Hotta and collaborators elegantly demonstrated that the synchronization of parasite development is lost in pinealectomized mice, whereas melatonin administration restored synchronicity in *P. chabaudi* [29].

**Figure 1:**
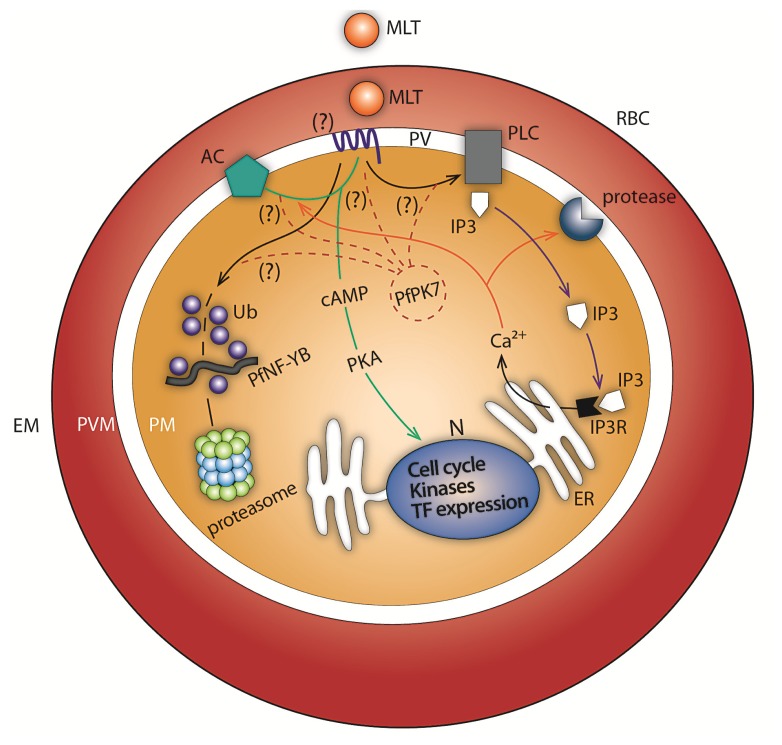
Melatonin molecular signaling pathways in *Plasmodium*. Melatonin (MLT) crosses the erythrocyte (RBC) surface membrane (EM) and the parasitophorous vacuolar membrane (PVM) into the parasitophorous vacuole (PV). *Plasmodium* senses MLT from the RBC and a cascade of signaling is initiated through an as yet unidentified melatonin receptor located in the parasite plasma membrane (PM) (?). Melatonin signaling activates phospholipase C (PLC) that induces the production of inositol triphosphate (IP_3_). IP_3_ is able to mobilize intracellular Ca^2+^ from the endoplasmic reticulum (ER) leading to a rise in cytosolic Ca^2+^ concentration through open ER-localized IP_3_-sensitive Ca^2+^ channels. In addition, melatonin signaling activates adenylyl cyclase (AC) producing an increase in cAMP level and further activation of protein kinase A (PKA). PKA is involved in controlling the balance of gene expression in the nucleus (N). Melatonin is also implicated in the activation of gene transcription for the UPS (ubiquitin proteasome system) machinery. Protein kinase 7 (PfPK7) seems to participate in the melatonin signaling pathway since parasites with a knockout of this kinase are not responsive to melatonin treatment, as measured by parasite intraerythrocytic stage distribution and activation of a subset of genes involved in UPS. However, how PfPK7 plays its role in melatonin signaling has not been identified yet. Most recently, it has been shown that melatonin increases ubiquitination of PfNF-YB transcription factor and increases its expression.

Melatonin precursors (serotonin, *N*-acetyl serotonin (NAS), and tryptophan) and derivatives (*N*(1)-acetyl-*N*(2)-formyl-5-methoxykynuramine (AFMK)) also modulate the parasite cycle and maintain the synchronicity of *P. falciparum* development [31,32]. In mammals, the asexual blood stage development of malaria parasites is variable between the species (based on 24, 48 or 72 h cycles), but is very synchronous. This synchronicity of parasite development is finely regulated *in vivo*, but if the parasite is cultured *in vitro* it is quickly lost. This effect can be explained by the absence of indoleamine hormone in the culture. Interestingly the peak of melatonin delivery *in vivo* overlaps with schizont maturation, merozoite release and RBC reinvasion in rodent parasites [29] suggesting that the *Plasmodium* parasite can sense melatonin levels. But how can melatonin modulate the parasite cell cycle? We have proposed that the presence of exogenous melatonin transduces a signal by coupling through an as yet unidentified *Plasmodium* melatonin receptor that activates phospholipase C (PLC). PLC induces the production of inositol triphosphate (IP_3_), which is a second messenger able to mobilize intracellular Ca^2+^ from subcellular compartments, leading to a rise in cytosolic Ca^2+^ concentration through open ER-localized IP_3_-sensitive Ca^2+^ channels in *P. falciparum* [29,33]. It was demonstrated that U73122 (PLC inhibitor) obstructed the cAMP increase indicating a cross talk between the Ca^2+^ and cAMP pathways in the parasite [34]. This melatonin signaling produces a complex cascade of events also involving a rise in cyclic AMP [34], as illustrated in Figure 1. In *Plasmodium*, cAMP signaling participates in several biological processes through activation of cAMP-dependent protein kinase A (PKA). In *P. falciparum* a downstream PKA response is important in parasite growth and the cell cycle, modulation of anion conductance, and erythrocyte invasion [35–37]. Recently, PKA was implicated in the control of parasite motility and cell invasion through phosphorylation of the glideosome components, myosin A and GAP45 and of Ca^2+^ dependent protein kinase-1 (CDPK1). It was suggested that the involvement of both PKA and CDPK1 provides a way of coordinating cAMP and Ca^2+^ fluxes to control motor activity and invasion [38]. The family of CDPKs such as CDPK1 provides a way to further coordinate the biological processes they control with Ca^2+^ fluxes in the cell [39].

The involvement of Ca^2+^ in the regulation of long-term cell adaptation through its ability to control gene expression has been considered a key feature in the biology of mammalian cells [40]. For *Plasmodium*, Ca^2+^ signaling has been reported by several labs to be crucial to cellular activities and the parasite life cycle [41–48].

## 3. Melatonin Modulates the Expression of a Subset of Genes of the Ubiquitin Proteasome System (UPS) in *P. falciparum*

The UPS machinery is highly conserved from yeast to human and is required for targeted degradation of protein in eukaryotic cells. The UPS pathway is characterized as a major catabolic process that results in the proteolysis of both cytosolic and nuclear proteins in an extra-lysosomal compartment—the proteasome [49–53]. As a result of its protein degradation activity, UPS plays a key role in the regulation of development, differentiation, proliferation, cell cycling, apoptosis, gene transcription, signal transduction, senescence, antigen presentation, inflammation, and the stress response [54–58].

Proteins are targeted for destruction first by covalent modification with ubiquitin, and then the ubiquitinated protein is guided to the 26S proteasome, a large multicatalytic multisubunit protease complex, which constitutes the central proteolytic machinery of the UPS. A group of three enzymes, E1, E2 and E3 is essential for the initial protein targeting during this process. E1 is an ubiquitin-activating enzyme that binds and activates ubiquitin through a thioester, in a step that requires energy from ATP. Then the ubiquitin is transferred to the active site cysteine of E2, an ubiquitin-conjugating enzyme, and finally the ubiquitination cascade ends with the covalent linkage created by E3 ubiquitin-protein ligases in which the carboxy-terminal glycine of ubiquitin forms an isopeptide bond to the side chain of a lysine residue of the target protein. The process of ubiquitin-mediated target protein delivery to the 26S proteasome may involve multiple cycles of ubiquitination resulting in the polyubiquitin chains that serve as a recognition signal for the 26S proteasome [49,50]. A subset of E3 ligases important in protein recognition involves the cullin and F-box proteins [59].

In *Plasmodium*, very little work has been done regarding the characterization of the parasite’s ubiquitin machinery [60]. However, due the important role of UPS in the cell, some studies have examined and predicted by *in silico* analysis a source of ubiquitin moieties in *Plasmodium*. For example, the polyubiquitin gene PF3D7_1211800 that codes for five conserved ubiquitin repeats, was identified [61–63]. The two ubiquitin fusion proteins PfUBS27 and PfUBL40 (PF14_0027 and PF13_0346, respectively) contain an ubiquitin moiety at their N-terminus [62]. Moreover, one conserved ubiquitin domain was identified in PF08_0067, a protein that is believed to be part of an endoplasmic reticulum-associated protein degradation (ERAD) 3-like pathway [63]. Further investigation, reveals other components of the *P. falciparum* ERAD system: one ubiquitin-activating E1 enzyme (UBA1), a ubiquitin-conjugating E2 enzyme (UBC), and a ubiquitin E3 ligase (*Plasmodium* HRD1). It was demonstrated that E1 and E2 localize to the cytosol whereas the E3 ubiquitin ligase is found within the ER membrane, consistent with their respective functions in the ERAD pathway [60].

Research in plant biology has revealed that the auxin hormone signal is sensed by components of the UPS that are commonly found in all eukaryotic cells. The auxin signaling pathway takes direct control of an E3 ligase and signals by promoting the ubiquitination and degradation of a set of transcriptional repressors [64,65]. Auxin is one of the most abundant natural plant molecules and its structure, as indole-3-acetic acid (IAA) is related to that of serotonin and melatonin. The biosynthesis of melatonin and IAA shares some common precursors, such as tryptophan and tryptamine [66,67]. Therefore, we were interested to investigate whether the melatonin signaling pathway acts on the *P. falciparum* UPS machinery. We demonstrated at the transcriptional level that melatonin treatment up-regulates genes coding for UPS components and that luzindole, a melatonin antagonist, inhibits the modulation of UPS transcription [68]. The transcript levels of E1 ubiquitin-activating enzyme, putative UBA1 and 2 (PFL1790w), E3 ubiquitin-protein ligase (Mal8P1.23), 26S proteasome regulatory subunit (RPN6, PF14_0025) and cullin-like protein (PF08-0094) increased after treatment with melatonin, thereby promoting ubiquitination of target proteins. Moreover, it was found that melatonin signaling involves an atypical protein kinase, PfPK7 [69,70], since melatonin treatment of *P. falciparum* parasites containing a disrupted *pk7* gene does not affect regulation of UPS genes. In addition, melatonin treatment of the *pk7* disrupted parasite did not affect the ratio of asexual stages and the increase in cytosolic Ca^2+^ was strongly diminished. Re-introduction of a functional copy of the *pk7* gene into the defective *pk7*-disrupted parasites restored the sensitivity to melatonin-induced alterations in UPS gene transcription. The importance and implications of melatonin signaling pathways modulating the UPS is a new area that deserves to be explored more fully since UPS is a crucial process central to cell development. Most recently, melatonin signaling was implicated in enhancing the ubiquitination of α-synuclein monomers and aggregates in the hippocampus of C57/BL6 mice. It was suggested that melatonin may exert its neuroprotection by inhibiting KA-induced autophagy and a subsequent mitochondrial loss as well as reducing α-synuclein aggregation by ubiquitination in the CNS [71].

## 4. Melatonin Signaling Modulates PfNF-YB Transcription Factor Expression in *P. falciparum*

In vertebrates, melatonin has a well-known regulatory role on the circadian rhythm, but beyond that it plays other biological functions in various cell types and peripheral tissues (for review see [72]). In different tissues and organs, melatonin has been described to act as a paracrine and also as an intracrine and autocrine agent with overall homeostatic functions and pleiotropic effects that include cell protection and survival. One of the roles of melatonin signaling is modulating gene regulation and expression. This indolamine is responsible for the control of transcription factor NF-YB-dependent genes including, among others, those coding for inflammatory mediators and antioxidant enzymes [73]. It has been demonstrated that melatonin signaling down-regulates the expression of Rex-1 and Oct4 transcription factors in murine embryonic stem cells [74].

The finding that melatonin controls the *Plasmodium* cell cycle and regulates transcription factor expression in vertebrates stimulated us to study the effect of the melatonin-induced signaling cascade upon regulation of gene expression in *Plasmodium*. Indeed, our group showed for the first time that the malarial transcription factor, PfNF-YB (PF11_0477), is regulated by melatonin signaling in the trophozoite stage of *P. falciparum*. Although PfNF-YB is expressed throughout the intraerythrocytic stages (ring, trophozoite and schizont forms), it was not examine whether PfNF-YB is ubiquitinated in the ring stage, since melatonin signaling does not affect this stage where the metabolic activity is lower compare to trophozoite and schizont forms. In addition, it was shown that melatonin affected the post-translational modification of PfNF-YB by ubiquitination. The melatonin cascade was able to increase PFNF-YB expression by two-fold and increase PFNF-YB ubiquitination in *P. falciparum* [75] (Figure 1). How melatonin signaling can modulate PfNF-YB expression through the cAMP cascade can be suggested since we observed that the cAMP second messenger can modulate PfNF-YB expression itself. Therefore, melatonin probably uses cAMP second messenger to regulate PfNF-YB expression. In contrast, melatonin signaling can upregulate mRNA expression of UPS related genes [68]. However, it has not been described yet whether melatonin signaling uses the same cascade to transcriptionally regulate genes or induce posttranslational protein modification. It was observed that melatonin signaling regulation of UPS genes is protein kinase 7 (PK7) dependent. Whether or not PK7 is required for regulation of PfNF-YB expression by melatonin deserves to be addressed.

PfNF-YB is the CCAAT-box-binding subunit B of the NF-Y complex and is conserved from yeast to human [76]. In mammals NF-YB binds to the CCAAT motif of several gene promoters during the G2/M phase of the cell cycle, such as genes coding for topoisomerase IIa, cyclin B1, CDC25C, E2F, CDC2 and thymidine kinase to modulate their expression [77–79]. Once the diverse functions of PfNF-YB in *P. falciparum* have been elucidated, the role played by PfNF-YB in melatonin signaling pathway modulation will be better understood. So far few other transcription factors have been described in *P. falciparum* and therefore the action of melatonin in modulation of other transcription factors has been very poorly investigated in *Plasmodium*. Melatonin may be involved in many other cellular pathways in addition to those already known in *Plasmodium*. We propose that the downstream molecular mechanism of melatonin signaling includes the modulation of gene expression for the UPS machinery.

Moreover, cAMP signaling differentially modulates PfNF-YB expression during intraerythrocytic stage in *P. falciparum*. In the ring stage a cAMP analogue decreases PfNF-YB expression while in trophozoite stage, the analogue increases PfNF-YB expression [75]. The PfNF-YB modulation by cAMP signaling can be explained by the fact that the cAMP response induces translocation of the PKA catalytic subunit to the nucleus where PKA modulates a variety of transcriptional regulators. The catalytic subunit gene of cAMP-dependent protein kinase (Pfpka-c) exists as a single copy in *P. falciparum*. Pfpka-c has been described as being expressed at high level in the pathogenic sexual stages and in the asexual parasites. PfPKA activity can be readily detected in schizonts and the parasite enzyme can be stimulated by cAMP [35].

## 5. Melatonin Blockers as Potential Antimalarial Drugs

Melatonin is synthesized in the pineal gland during the night mainly due the stimulation of the enzyme, arylalkylamine *N*-acetyltransferase (AANAT) in response to the photoneuroendocrine system. For melatonin biosynthesis, the amino acid tryptophan is converted into serotonin, which is then metabolized into melatonin by two different enzymes: AANT and hydroxyindole-*O*-methyltransferase (HIOMT). AANT *N*-acetylates serotonin to form *N*-acetylserotonin, which in turn undergoes *O*-methylation by HIOMT enzyme, to form melatonin [80,81]. In addition, the SCN regulates melatonin production and AANAT activity by a norepinephrine neurotransmitter that acts in a circadian manner during the night. The norepinephrine activates the β1- and α1-adrenergic receptors. The first, the β1-adrenergic receptor, increases the intracellular cAMP resulting in activation of PKA, both of which stimulate AANAT and melatonin generation. The second, α1-adrenergic receptor activation causes an increase in Ca^2+^ influx by releasing Ca^2+^ ions from intracellular stores in pinealocyte cells [82,83]. In exposure to bright light AANAT is degraded and consequently melatonin production is inhibited [84].

During the night the neurohormone melatonin is produced and is spread throughout the body via the circulation. Via the bloodstream melatonin reaches the cells of various organs and is found distributed in lymphocytes, the thymus, skin, the eyes, the gastrointestinal tract, and the bone marrow (reviewed in [85]). Although it freely diffuses through all biological membranes, melatonin internalization is also receptor-mediated. Four receptors have been described that enhance melatonin internalization into cells. Melatonin binds with high affinity to melatonin receptor MT_1_, with a dissociation constant (Kd) <200 pM [86]. The MT_2_ receptor is very similar to MT_1_ sharing 60% amino acid sequence identity [87]. A third melatonin-related receptor, known as GPR50, shares 45% amino acid sequence identity with MT_1_ and MT_2_[88] and seems to form a heterodimers with MT_1_ and MT_2_[89]. The MT_3_ melatonin receptor (previously called ML-2) binds to melatonin with lower affinity (Kd 0.9–10 nM). MT_1_ and MT_3_ receptors are coupled to G proteins [90]. Melatonin also interacts with nuclear receptors belong to the RZR/ROR subfamily which by alternative splicing creates the RORα, RZRβ and RORγ receptors [91].

How can understanding *Plasmodium* melatonin signaling contribute to the discovery of new antimalarial drugs? This idea is based on the findings that melatonin contributes to the control of parasite replication and survival and therefore these pathways may be targeted and blocked. Melatonin antagonists have been identified, and one of the most powerful is luzindole [92]. Luzindole has a higher affinity for MT_2_ than MT_1_, by approximately 11- to 25-fold [90,93]. This antagonist has been used by several researchers to show the effect of melatonin, for instance describing the blockage of dopamine release in rabbit retina [94]. Luzindole has been described as abolishing the action of melatonin on *Plasmodium* [32,68,95,96]. However, it is still unknown whether the *Plasmodium* melatonin receptor works through a GPCR signaling mechanism. Luzindole blocks melatonin action and interferes with synchronization of both *P. falciparum* and *P. chabaudi in vitro*. Melatonin signaling activates PLC leading to an increase of Ca^2+^ and IP_3_ generation [29,30,33], and this effect is abolished in the presence of both luzindole and *U73122* (an inhibitor of phospholipase C). Melatonin directly modulates the parasite-host interaction, for example in mice melatonin suppresses hepatocyte apoptosis during *Plasmodium yoelii* infection by preventing oxidative stress. The mitochondrial pathway of apoptosis, which plays the critical role, is blocked by melatonin [97]. However, asynchronous *P. berghei* and *P. yoelii* are insensitive to the effect of melatonin, both with respect to Ca^2+^ flux and cell cycle modulation [98].

Parasite synchronization with the circadian rhythm is an evolutionary adaptation [99,100] to evade the immune system and to deliver gametocytes at the optimal time for uptake by feeding mosquitoes [9]. In addition, it was shown that when parasites lose synchronicity the protection provided by classical antimalarial drugs is enhanced at suboptimal doses [95]. These represent new avenues of research for the development of new combinations of pharmacologic drugs against malaria.

## 6. Conclusions

The malaria parasite is a very versatile pathogen allowing it to escape from the immune system and kill many people each year. New malaria infections are possible, for example the emergence in Malaysia and surrounding countries of human infection caused by a virulent parasite, *P. knowlesi* [101]. It is important to focus on elucidation of the basic biology of the parasite-host interaction and the mechanisms of how *Plasmodium* parasites evade the host immune response and develop resistance to antimalarials. Understanding drug resistance and its prevention is at the top of WHO priorities to fight malaria, complementing parasite elimination programs based on pharmacological drugs and insect control. Melatonin has been reported to modulate the *Plasmodium* cell cycle, but melatonin signaling pathways are complex and activate a subset of genes from the UPS machinery (ubiquitin proteasome system), as well as modulating transcription factor expression. This probably occurs in *Plasmodium* through activation of second messengers, including Ca^2+^, cAMP and IP_3_. Therefore, melatonin signaling pathways represent new ways to understand, dissect and combat the malaria parasite, encompassing the idea of using melatonin antagonists as antimalarial drugs (see reviews [85,102]).
